# Genome-wide identification of the tobacco *GDSL* family and apical meristem-specific expression conferred by the *GDSL* promoter

**DOI:** 10.1186/s12870-021-03278-x

**Published:** 2021-10-30

**Authors:** Jing Lv, Chang-Bo Dai, Wei-Feng Wang, Yu-He Sun

**Affiliations:** 1 Tobacco Research Institute, Chinese Academy of Agricultural Sciences, Qingdao, 266101, China; 2 Key Laboratory for Tobacco Gene Resources, State Tobacco Monopoly Administration, Qingdao, 266101 China; 3 Graduate School of Chinese Academy of Agricultural Sciences, Beijing, 100081 China

**Keywords:** *GDSL* gene family, Transcriptome data, Axillary bud development, Tissue-specific promoter

## Abstract

**Background:**

GDSL esterases/lipases are a large protein subfamily defined by the distinct GDSL motif, and play important roles in plant development and stress responses. However, few studies have reported on the role of GDSLs in the growth and development of axillary buds. This work aims to identify the GDSL family members in tobacco and explore whether the *NtGDSL* gene contributes to development of the axillary bud in tobacco.

**Results:**

One hundred fifty-nine *GDSL* esterase/lipase genes from cultivated tobacco (*Nicotiana tabacum*) were identified, and the dynamic changes in the expression levels of 93 of these genes in response to topping, as assessed using transcriptome data of topping-induced axillary shoots, were analysed. In total, 13 *GDSL* esterase/lipase genes responded with changes in expression level. To identify genes and promoters that drive the tissue-specific expression in tobacco apical and axillary buds, the expression patterns of these 13 genes were verified using qRT-PCR. GUS activity and a lethal gene expression pattern driven by the *NtGDSL127* promoter in transgenic tobacco demonstrated that *NtGDSL127* is specifically expressed in apical buds, axillary buds, and flowers. Three separate deletions in the *NtGDSL127* promoter demonstrated that a minimum upstream segment of 235 bp from the translation start site can drive the tissue-specific expression in the apical meristem. Additionally, *NtGDSL127* responded to phytohormones, providing strategies for improving tobacco breeding and growth.

**Conclusion:**

We propose that in tobacco, the *NtGDSL127* promoter directs expression specifically in the apical meristem and that expression is closely correlated with axillary bud development.

**Supplementary Information:**

The online version contains supplementary material available at 10.1186/s12870-021-03278-x.

## Background

In tobacco field production, the floral parts along with undeveloped leaves in the upper part of the plants are removed before harvest to enhance growth and development of the remaining leaves, in a process known as topping. The control of tobacco (*Nicotiana tabacum*) apical and axillary bud development before and after topping is a research focus in tobacco agriculture. In most plants, the shoot apical meristem plays a vital role in plant development [1]. Lateral branches develop from the axillary buds and significantly impact the biomass, morphology and quality of tobacco morphological and biomass [2] In addition, apical and axillary bud outgrowth are under homeostatic control [3].

Currently, two types of genes are known to be involved in axillary bud formation and regulation. One type is involved in the initiation of the axillary meristem and includes GRAS, MYB, and NAC transcription factors. The other type is involved in the regulation of axillary bud growth and includes F-box protein, and knott-like and SPL transcription factors. GDSL lipase is a hydrolytic enzyme with a conserved GDSL domain (pfam PF00657) at the N terminus of the protein, found widely across both prokaryotes and eukaryotes [4]. Plant GDSL lipases form a large gene family, and members have been identified in *Arabidopsis* (105) [5], *Oryza sativa *(114) [6], six Rosaceae genomes (597) [7], and *Brassica rapa* L. (121) [8]. Members of the plant *GDSL* family form three large subfamilies (I, II, and III) in the phylogenetic tree, and great structural and functional diversity exists among them [9]. GDSL lipases regulate lateral root growth [10], embryo growth [11], seed and pollen development [12], and disease and stress resistance [13, 14]. Limited studies have examined the role of GDSL in the growth and development of axillary buds.

To date, many promoters of interest have been used for the genetic improvement of tobacco [15, 16]. The production of cellulase in tobacco driven by the *RbcsK-1A* promoter served as a foundation for the commercialization of bioethanol production [17]. In addition, the expression of isopentenyl transferase in tobacco under the control of the stress-inducible promoter *rd29A* significantly enhances tolerance to salt stress [18], and similarly, various promoters have been effective in increasing cold resistance [19], drought-stress tolerance [20], and disease resistance [21] in tobacco. Moreover, a specific promoter combined with a toxic protein gene, such as Diphtheria toxin A chain (*DTA*), which can ribosylate the elongation factor-2 (EF2) translation initiation factor and subsequently inhibit all protein translation, is effective in controlling tissue-specific expression [22, 23]. These promoters are tissue-specific and can therefore control gene expression in particular cells or tissues to avoid the unnecessary waste associated with constitutive expression. In the aerial parts of dicotyledons, meristem tissues are found in the apical and axillary buds, where meristem-specific promoters can drive genes associated with plant growth and development. Modification of apical and axillary bud growth in plants is possible through genetic engineering [24]. The creation of early flower materials in *Arabidopsis* through *FT* overexpression driven by meristem-specific *KNAT1* gene has been reported [25], and reporter genes under the control of apex-specific promoters have been used to characterise apex behaviour [26].

In field production, suckercides are extensively applied to tobacco after topping to inhibit the growth of axillary buds [27], which requires time, effort, and resources to carry out. Further verification of apical meristem-specific genes and their promoters may provide alternate ways to control the growth of axillary buds after topping in tobacco. Differential gene expression data based on RNA sequencing (RNA-seq) from untopped and topped tobacco plants have been analysed to determine the global changes in gene expression in response to topping [28]. In this study, we identified the complete set of GDSL proteins in tobacco using the reannotated transcriptome data of tobacco [28]. Compared with the transcriptome data annotated by the 2014 version of tobacco genome, the transcriptome data annotated by the 2017 version has a higher read matching probability (Table 1). Use of the new version of the annotation is conducive to a more comprehensive and systematic analysis and identification of the *GDSL* gene family.

**Table 1:** Statistics of clean reads in the transcriptomes annotated by 2017 version of tobacco genome and 2014 version of tobacco genome seperately

sample	2017 clean_bases	2014 clean_bases	2017 Total mapped (%)	2014 Total mapped (%)	2017 Uniquely mapped (%)	2014 Uniquely mapped (%)	2017 Spliced reads (%)	2014 Spliced reads (%)
NY1	7.57G	7.65G	95.62	89.70	87.97	87.31	32.23	31.53
NY2	8.68G	8.73G	95.24	89.64	87.85	87.73	32.70	32.17
NY3	7.09G	7.17G	95.64	90.05	88.13	87.71	32.63	32.00
TY11	7.38G	7.41G	95.07	89.05	87.66	87.18	33.43	32.83
TY12	9.08G	9.14G	95.26	89.10	87.72	87.07	33.32	32.64
TY13	8.57G	8.65G	95.45	89.56	87.88	87.25	33.59	32.91
TY41	7.25G	7.29G	94.94	88.15	87.43	86.24	32.66	31.90
TY42	8.43G	8.43G	94.59	87.90	87.15	86.40	32.51	31.86
TY43	7.56G	7.62G	94.84	88.24	87.43	86.10	33.09	32.23
TY51	8.85G	8.91G	94.73	88.17	87.26	86.09	32.83	31.99
TY52	7.93G	7.98G	94.53	87.90	86.95	85.84	31.99	31.17
TY53	10.02G	10.08G	95.08	88.69	87.47	86.67	32.87	32.15

A comprehensive analysis of the expression profile of *GDSL* genes utilizing the reanalysed transcriptome of topping-induced axillary shoots in *N. tabacum* was conducted to identify genes that respond to topping induction and promoters that drive tissue-specific expression in the apical meristem and axillary buds. Specificity of the promoters and the correlations between the *GDSL* genes and axillary bud development were explored.

## Results

### Identification and phylogenetic analysis of *GDSL* family members in tobacco

**Fig. 1:**
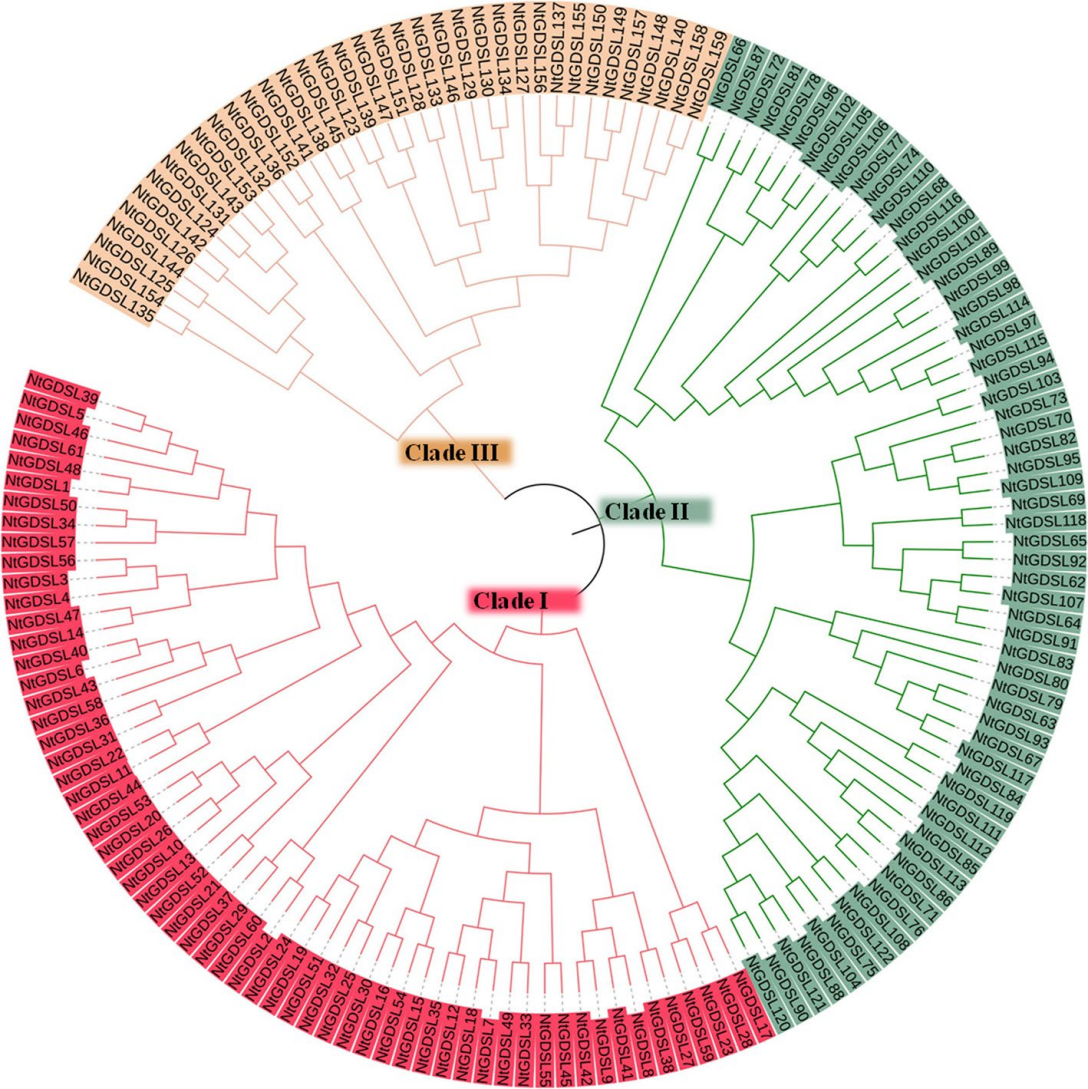
Phylogenetic relationship of *GDSL* gene family in tobacco. The GDSL proteins were divided into three distinct subgroups

**Fig. 2:**
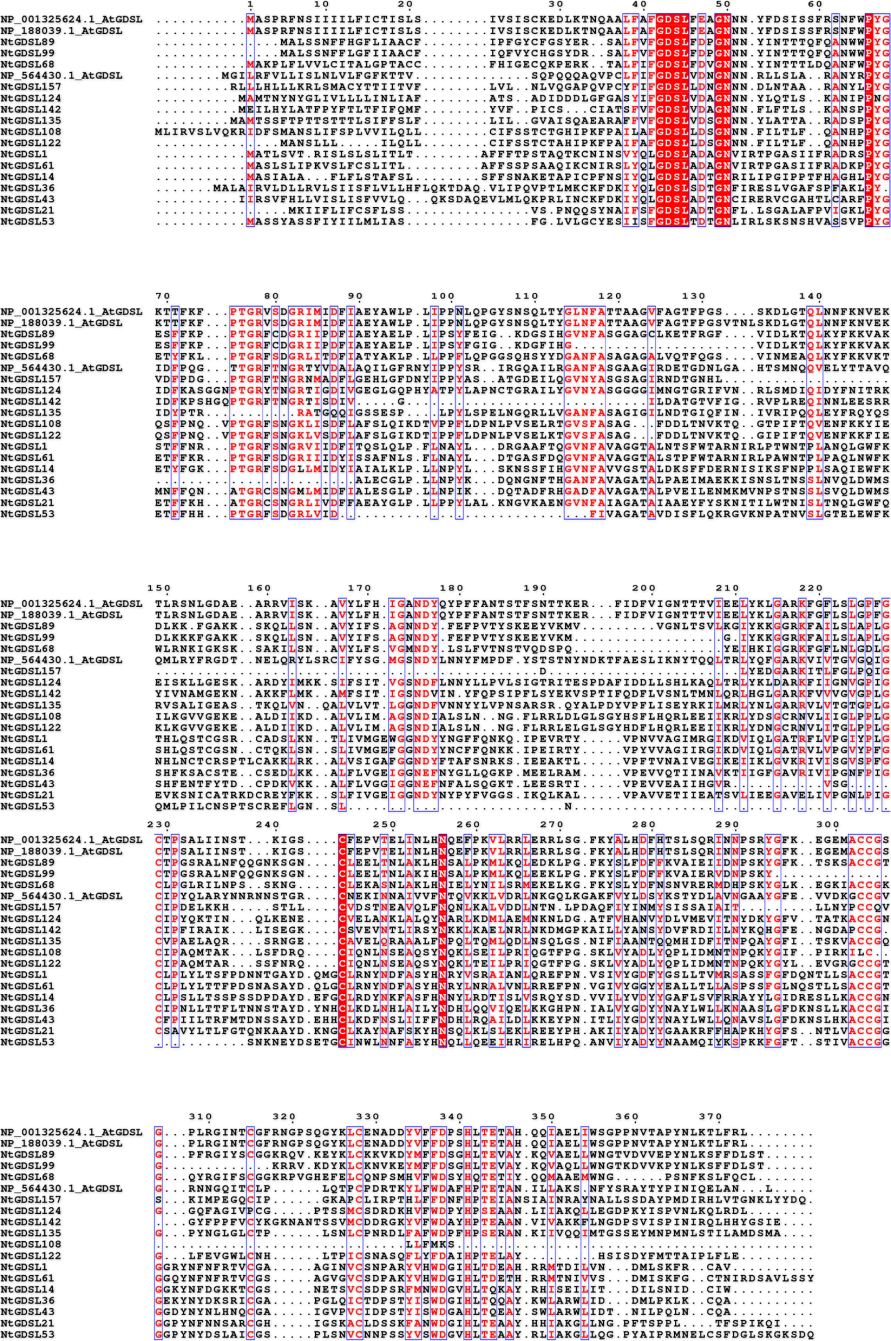
Conserved motifs of representative *GDSL* in tobacco and Arabidopsis. Representative GDSL proteins in tobacco and Arabidopsis obtain typical GDSL conserve domains

To identify *GDSL* genes in tobacco, an HMM search was performed against the reannotated tobacco protein sequences using the Pfam GDSL domain (PF00657) as the query. Newly identified entries were used as queries to carry out a BLASTP-based search against the 2017 annotations to the tobacco genome. After manually removing redundant hits, the resulting sequences were further analysed with both Pfam (https://pfam.xfam.org/) and SMART (http://smart.embl.de/) to ensure the presence of the GDSL domain. A total of 159 non-redundant GDSL family proteins were identified in tobacco, which was more than has been identified in any other species. Because *N. tabacum* is an allotetraploid, the large number of *GDSL* genes is expected. The gene name, gene ID, protein isoelectric point, and molecular weight of 159 *GDSL* members are listed in Supplementary Table S1. To better understand the evolutionary relationships among *GDSL* members, an unrooted phylogenetic tree was constructed using the full-length sequences of the 159 GDSL proteins (Fig. 1). Based on the neighbour-joining phylogenetic analysis, the GDSL proteins were divided into three distinct subgroups: Clades I, II, and III, which contained 61, 61, and 37 *GDSL* genes, respectively. The sequences of the *GDSL* family members in tobacco are listed in Supplementary Table S3. Alignment results of representative 16 GDSL proteins in tobacco and 3 GDSL proteins in *Arabidopsis* showed they all contained typical GDSL conserved domains (Fig. 2).

### *GDSL* genes involved in topping treatment

**Fig. 3:**
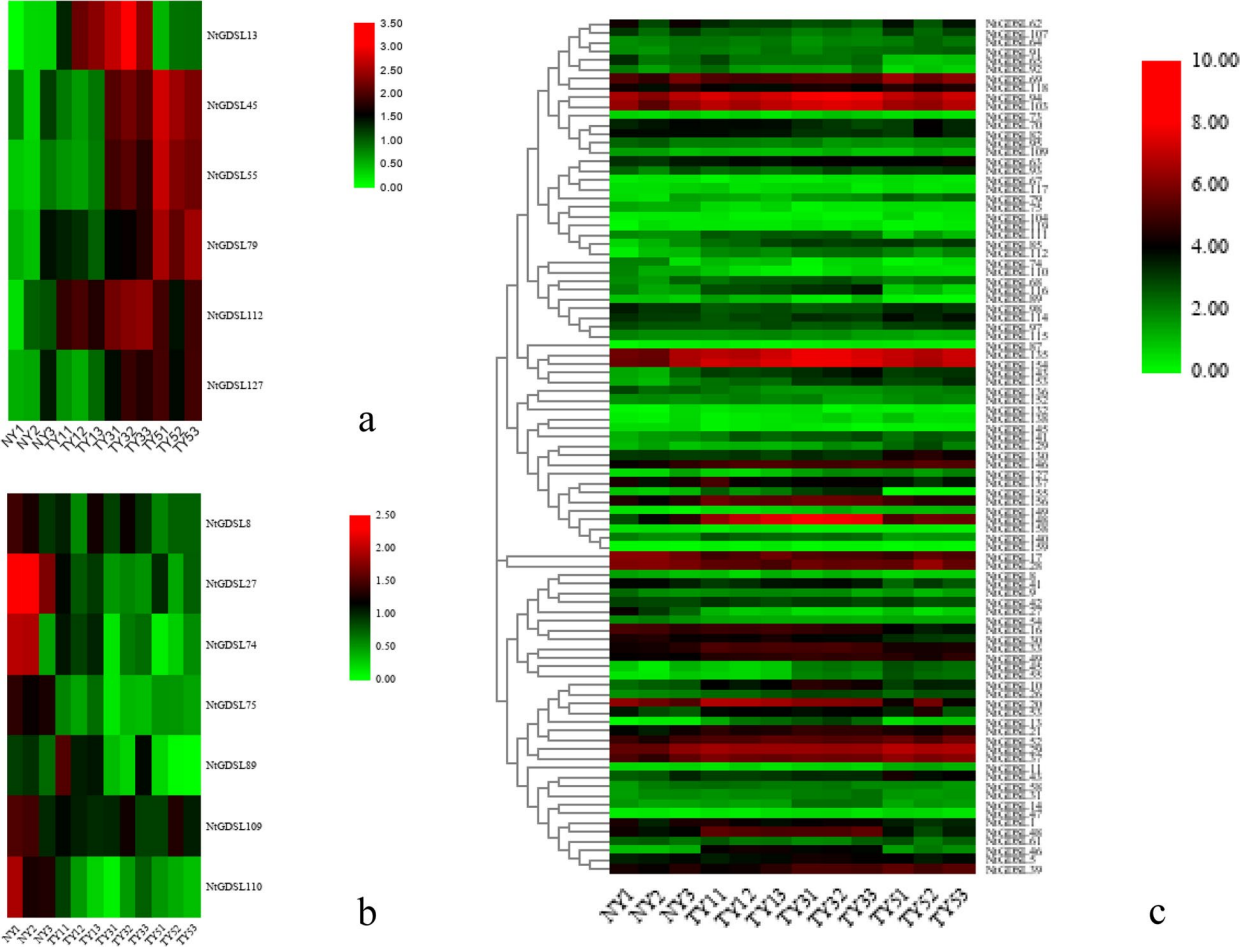
Heatmap and hierarchical clustering of representative *GDSL* gene members before and after topping. Changes in expression levels were displayed from green(down-regulated) to red (up-regulated), as shown in the color gradient. NY: Axillary buds on 1st leaf before topping, TY1:Axillary buds on 1st leaf 1 day after topping, TY3:Axillary buds on 1st leaf 3 days after topping, TY5:Axillary buds on 1st leaf 5 days after topping. Ninety-three genes showed changes in expression, while 6 and 7 candidate genes were significantly up-regulated and down-regulated twofold or more after topping, respectively.**a**: Heatmap of 6 candidate *GDSL* genes that are up-regulated after topping, **b**: Heatmap of 7 candidate *GDSL* genes that are down-regulated after topping. **c**: Heatmap of all the 93 *GDSL* genes

**Fig. 4:**
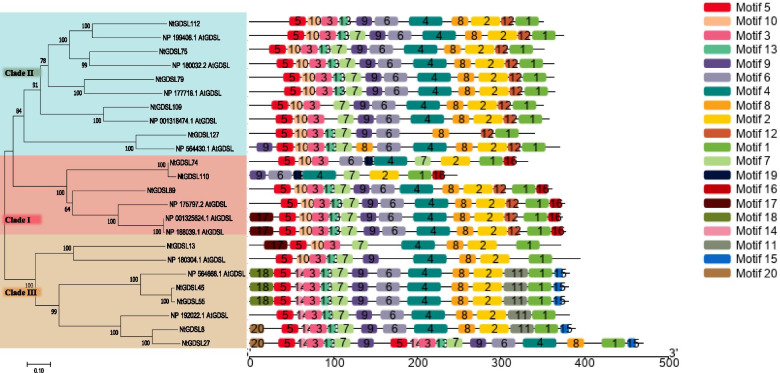
Phylogenetic relationship of 13 candidate genes in tobacco and 11*GDSL* genes in Arabidopsis. The same subfamilies share similar motif organization, some unique motif exist in different clades

We analyzed the expression profiles of the 159 *GDSL* genes before and 1–5 days after topping from the RNA-seq data, and found that 93 of these genes showed changes in expression (Fig. 3c). The axillary buds on the first leaf before topping (NY), the axillary buds on the first leaf 1 day after topping (TY1), the axillary buds on the first leaf 3 days after topping (TY3), and the axillary buds on the first leaf 5 days after topping (TY5) were analyzed. From the transcriptome data, we selected 6 and 7 candidate genes that were significantly up- and down-regulated, respectively, twofold or more after topping (Fig. 3a, Fig. 3b). An unrooted phylogenetic tree was constructed with these 13 GDSL proteins and 11 representative GDSL proteins in *Arabidopsis*, and the conserved motifs in 24 GDSLs were predicted via MEME (Fig. 4). In total, 20 conserved motifs (motif 1 to motif 20) were identified in the GDSL proteins. The width these motifs ranges from 8 to 41 amino acids, the e-value is 2e-88, and the same subfamilies share similar motif organization. Some motifs are unique to a certain clade: for instance, motifs 11, 14, 15, 18, and 20 are only found in Clade III, while motifs 16 and 19 only exist in Clade II, whereas motif 12 is shared by Clades I and II and absent in Clade III. Other motifs are common and regularly arranged in the GDSL proteins.

**Fig. 5:**
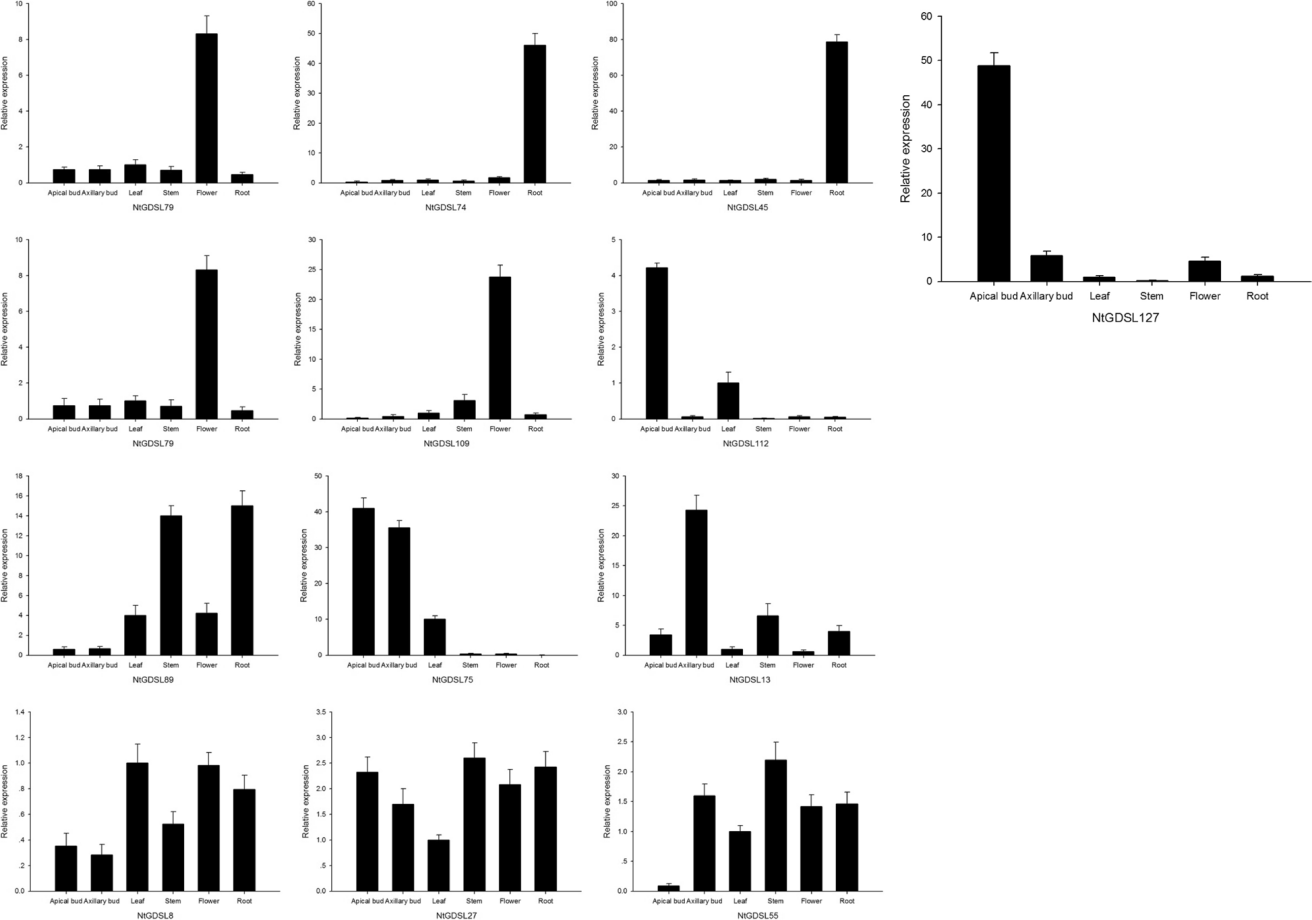
Expression levels of the 13 candidate genes. From left to right: apical bud, axillary bud, leaf, stem, flower, root. X axis represents the types of tissue, Y axis represents the relative expression quantity of *NtGDSL .* One GDSL esterase/lipase gene *NtGDSL127* exhibited apical meristem specificity

To further explore the expression pattern of these topping-induced genes, tissue-specific expression of these 13 *GDSL* genes was examined with qRT-PCR (Fig. 5). *NtGDSL45*, *NtGDSL74,* and *NtGDSL110* are mainly expressed in the roots; *NtGDSL79* and *NtGDSL109* are chiefly expressed in flowers. A notable result is that the gene *NtGDSL127* exhibited apical meristem specificity, while the remaining 7 genes had no observed tissue-specific expression. In order to further study the functions and possible applications of *GDSL* genes in apical bud development, we selected *NtGDSL127* for further analysis.

### Hormone experiment

**Fig. 6:**
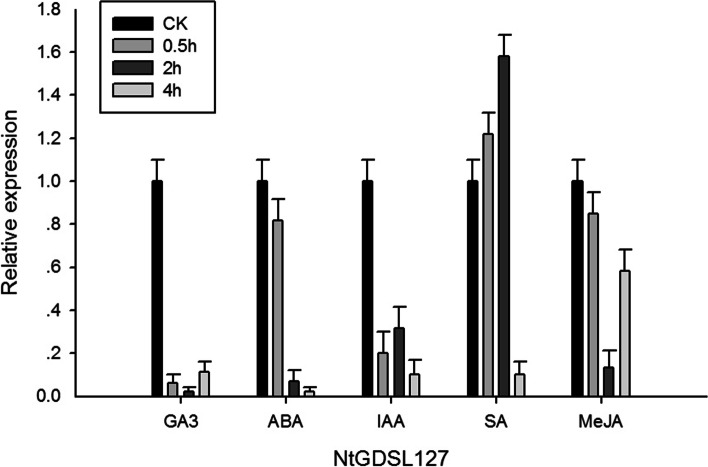
Expression level of the *NtGDSL127* under different hormone treatments. X axis represents the types of hormone application, Y axis represents the relative expression qualntity of *NtGDSL127 . NtGDSL127* may involve in the bud development of tobacco by responding to different hormone treatments

Based on previous reports that many *GDSL* genes can respond to phytohormones, we hypothesized that *NtGDSL127* could also be involved in plant organ development by responding to phytohormones. The results of fluorescence quantitative PCR supported this hypothesis (Fig. 6). Down-regulation of *NtGDSL127* was observed after GA3 and ABA application. At 2 h after the salicylic acid treatment, expression of *NtGDSL127* reached a peak and then declined, while expression of *NtGDSL127* reached a minimum at 2 h after the methyl jasmonate treatment and then increased. Expression of *NtGDSL127* after the indole-3-acetic acid treatment was irregular. The results indicate that *NtGDSL127* may be involved in bud development in tobacco.

### Isolation and sequence analysis of the *NtGDSL127* promoter

**Fig. 7:**
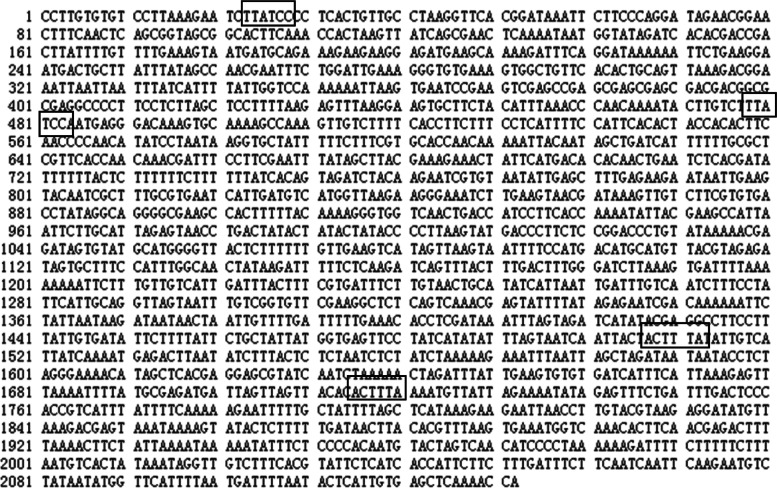
Sequence information of *NtGDSL127* promoter. The promoter includes several regulatory elements that may be involved in tissue-specific expression regulation. SRE-like sequences: TTATCC, NTBBF1ARROLB: ACTTTA

A promoter region 2,132-bp upstream of the translation initial codon (ATG) of *NtGDSL127* was isolated from the tobacco variety ‘honghuadajinyuan’ (Fig. 7). This fragment was designated *P*_*NtGDSL127*_ and submitted to PLACE databases to detect potential regulatory elements involved in the regulation of expression specificity. Several potential regulatory elements were identified within this promoter (Table 2). The promoter includes basal regulatory elements, such as a TATA-box and GATA-box. It also contains cis-elements involved in hormone induction, such as the ABA-responsive element, the low temperature-responsive element, and the GA3-responsive element. Several regulatory elements that may be involved in green tissue-specific expression regulation were also detected, such as the G-box, A-box, and embryo- and endosperm-specific motifs. Two copies of TTATCC and two copies of ACTTTA (Fig. 7) were correlated with meristem-specific expression. The TTATCC element is required for axillary bud outgrowth [29], while the ACTTTA motif is a tissue-specific expression element [30].

**Table 2:** Putative cis-elements identified in the P_*NtGDSL127*_ sequence

Motif	Sequence	Distance from ATG	Function
DPBFCOREDCDC3	ACACNNG	− 2118, − 1821, − 1270, − 946, − 460, −328, − 292	Target site for trans-acting StDof1 protein controlling guard cell-specific gene expression
DOFCOREZM	AAAG	− 2117, − 1957, − 1942, − 1921, − 1854, − 1844, − 1820, − 1638, − 1630, − 1624,-1450, − 1269, − 1220, − 945,-565, − 459,-353, − 327, −291, − 276, − 150	Core site required for binding of Dof proteins in maize
ARR1AT	NGATT	− 1919, − 1860,-1477,-986, − 941, − 912, − 899, − 871, − 744, − 489, − 434, − 384, − 148, − 79, − 31	“ARR1-binding element” found in Arabidopsis
IBOXCORE	GATAA	− 2079, − 1910, − 1272, − 762, − 726, − 546, − 260	I box, Conserved sequence upstream of light-regulated genes of both monocots and dicots
SREATMSD	TTATCC	− 2109, − 1654	Identification of cis-elements that regulate gene expression during initiation of axillary bud outgrowth in Arabidopsis
GATABOX	GATA	−2079,-2063, − 1910, − 1415, − 1272,-1091, − 1013, − 762, − 726,-685, − 546, − 299, − 260	Required for high level, light regulated, and tissue specific expression
MYBST1	GGATA	− 2080,-2064, − 1911, − 300	function as a transcriptional activator
GTGANTG10	GTGA	− 1847,-1772, − 1317, − 1255, − 942,-900, − 687, − 660, − 473, − 242, − 14	cis-regulatory element in the promoter of the tobacco late pollen gene g10
CACTFTPPCA1	YACT	− 2100, − 2030,-2020,-1830, − 1663,-1585, − 1577, − 1406, − 1231, − 1145,-1140,-1072, − 965, − 907, − 629, − 626, − 586, − 418, − 270, − 228,-171, − 126, − 22	cis-Regulatory elements for mesophyll-specific gene expression in the C4 plant *Flaveria trinervia*
MYBCORE	CNGTTR	− 2098,-1825	Binding site for all animal MYB and at least two plant MYB proteins ATMYB1 and ATMYB2, both isolated from Arabidopsis
RAV1AAT	CAACA	− 1671, − 1566,-1527, − 1484, − 164	Binding consensus sequence of Arabidopsis transcription factor, RAV1
REBETALGLHCB21	CGGATA	− 2081	Required for phytochrome regulation
GT1CONSENSUS	GRWAAW	−2079,-1910,-1814, − 1289,-1272, −762, − 726,-546, − 529, − 400	Consensus GT-1 binding site in many light-regulated genes
BOXIINTPATPB	ATAGAA	− 2062	“Box II” found in the tobacco plastid atpB gene promoter
MYBCOREATCYCB1	AACGG	− 2058	cis-acting element involved in cell cycle phase-independent activation
CAREOSREP1	CAACTC	− 2047	cis-acting elements necessary and sufficient for gibberellin-upregulated proteinase expression in rice seeds
MYB1AT	WAACCA	− 2024,-5	transcriptional activators in abscisic acid signaling
CARGCW8GAT	CWWWWWWWWG	−2000, − 1796, −624,-416, − 124, − 52,-39	Binding site selection for the plant MADS domain protein AGL15
TATABOX5	TTATTT	− 1970, − 1883,-363	cis elements and trans-acting factors affecting regulation of a nonphotosynthetic light-regulated gene
POLASIG3	AATAAT	− 1997, − 1342,-543	consensus sequence for plant polyadenylation signal
S1FBOXSORPS1L21	ATGGTA	− 1993	negative cis-element conserved in plastid-related genes
DRE2COREZMRAB17	ACCGAC	− 1976	Regulatory elements in vivo in the promoter of the abscisic acid responsive gene rab17 from maize
DRECRTCOREAT	RCCGAC	−1976	Core motif of transcription activators that function in drought-, high-salt- and cold-responsive gene expression
CBFHV	RYCGAC	−1976,-786	Binding site of barley dehydration-responsive element (DRE) binding proteins
LTRECOREATCOR15	CCGAC	− 1975	Core of low temperature responsive element (LTRE) of cor15a gene in Arabidopsis
ANAERO1CONSENSUS	AAACAAA	− 1966	motifs in promoters of anaerobically induced genes of different plant species
POLLEN1LELAT52	AGAAA	− 1944,-1448, − 563, − 401	required for pollen specific expression
NODCON1GM	AAAGAT	−1921, −150	nodule specificity of cis-acting regulatory elements in the soybean leghemoglobin
WBOXHVISO1	TGACT	− 1890,-1151,-960,-379	sugar-responsive element
WRKY71OS	TGAC	−1890, − 2828, − 1254, − 1206, − 1151, − 1122, − 1033, − 960, − 379	transcriptional repressor of the gibberellin signaling pathway in aleurone cells
WBOXNTERF3	TGACY	− 2374,-1254,-1206,-1151,-1122, − 960, − 379	involved in activation of ERF3 gene by wounding
TATABOX2	TATAAAT	−123	Sequences responsible for the tissue specific promoter activity of a pea legumin gene
EECCRCAH1	GANTTNC	− 1868, − 1476, − 911, − 658, − 391,-147	cis-acting elements and DNA-binding proteins involved in CO2-responsive transcriptional activation of Cah1
INRNTPSADB	YTCANTYY	− 1600,-41	Light-responsive transcription of psaDb
CAATBOX1	CAAT	− 1649,-1515, − 1329, − 863,-633, − 502,-176, − 70, − 66	CAAT promoter consensus sequence
SORLIP1AT	GCCAC	− 1233	involved in the network of phytochrome A-regulated gene expression
MYB2AT	TAACTG	− 889	MYB recognition sequence
MYB2CONSENSUSAT	YAACKG	− 1429,-1210,-889	MYB recognition site found in the promoters of the dehydration-responsive gene rd22 and many other genes in Arabidopsis
POLASIG1	AATAAA	− 280,-196	Putative polyadenylation signals in nuclear genes of higher plants
CCAATBOX1	CCAAT	− 1650	“CCAAT box” found in the promoter of heat shock protein genes
CGACGOSAMY3	CGACG	− 1742, − 1739	cis-element required for rice alpha-amylase Amy3D expression during sugar starvation
NODCON2GM	CTCTT	− 1719,-1404, − 1070, − 268	nodulin consensus sequences
PYRIMIDINEBOXOSRAMY1A	CCTTTT	− 1710	Gibberellin-respons cis-element of GARE
L1BOXATPDF1	TAAATGYA	− 1683	a cis-regulatory element for L1 layer-specific gene expression
TBOXATGAPB	ACTTTG	− 964,-958	reductions of light-activated gene transcription
-300ELEMENT	TGHAAARK	− 1634	an enhancer element for the endosperm-specific expression of high molecular weight glutenin
CARGATCONSENSUS	CCWWWWWWGG	− 1558	component of the MADS-box flowering-time gene
CPBCSPOR	TATTAG	−405	Cis-Element Exhibiting Cytokinin-Dependent Protein Binding in Vitro
GT1GMSCAM4	GAAAAA	− 1543,-1400, − 983, − 140	Plays a role in pathogen- and salt-induced SCaM-4 gene expression
RHERPATEXPA7	KCACGW	− 1419,-517, − 106	Root Hair Cell-Specific cis-Element
SEF4MOTIFGM7S	AACAAAC	− 1483	minimal cis-element requirements for endosperm-specific gene expression
MYCCONSENSUSAT	CANNTG	−1429,-1210, − 1001	MYC recognition site found in the promoters of the dehydration-responsive gene rd22 and many other genes in Arabidopsis
CIACADIANLELHC	CAANNNNATC	−1429	Region necessary for circadian expression of tomato Lhc gene
CURECORECR	GTAC	− 1332, − 310, − 172	core of a CuRE (copper-response element) found in Cyc6 and Cpx1 genes in Chlamydomonas
NTBBF1ARROLB	ACTTTA	− 625, − 417	NtBBF1 binding site in *Agrobacterium rhizogenes* rolB gene; Required for tissue-specific expression and auxin induction
QELEMENTZMZM13	AGGTCA	−1254	pollen-specific
WBOXATNPR1	TTGAC	− 961, − 380	WRKY binding sites
ACGTABOX	TACGTA	−1021, − 309	sugar repression

### GUS verification of *P*_*NtGDSL127*_ and 5′ deletion promoters in transgenic tobacco

**Fig. 8:**
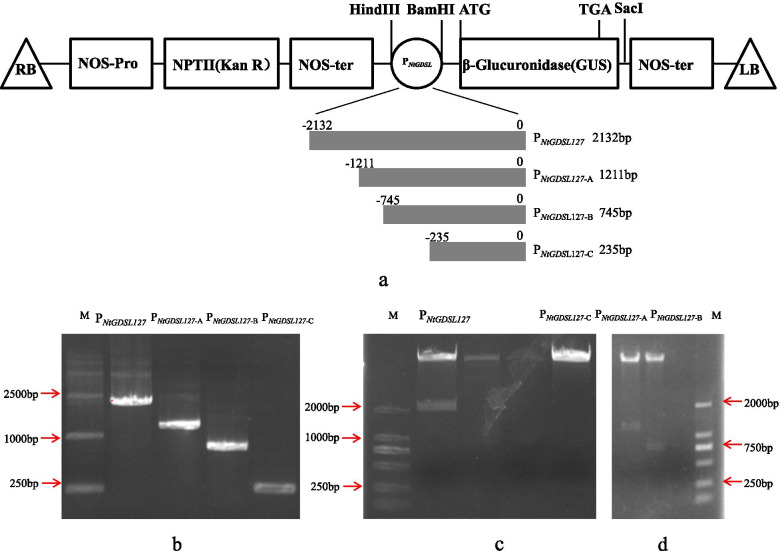
Construction of recombinant vectors. **a**: Schematic map of the *P*_*NtGDSL127*::_*GUS* recombinant vectors. The promoter fragments were inserted at the *Hind*III and *Bam*HI sites of PBI121. The *GUS* gene was driven by the *P*_*NtGDSL127*_ promoter and the 5′ deletion promoters seperately. **b**: Agarose gel separation of PCR product of *P*_*NtGDSL127*,_ *P*_*NtGDSL127-A*_, *P*_*NtGDSL127-B*_ and *P*_*NtGDSL127-C*_, **c**: Enzymatic digestion of the recombinant plasmid *P*_*NtGDSL127*_ and *P*_*NtGDSL127-C*_ using *Hind*III and *Bam*HI. **d**: Enzymatic digestion of the recombinant plasmid *P*_*NtGDSL127-A*_ and *P*_*NtGDSL127-B*_ using *Hind*III and *Bam*HI. 22(*P*_*NtGDSL127::*_*GUS*), 25(*P*_*NtGDSL127-A::*_*GUS*), 27(*P*_*NtGDSL127-B::*_*GUS*), and 30(*P*_*NtGDSL127-C::*_*GUS*) independent transformants of each construct were obtained seperately

**Fig. 9:**
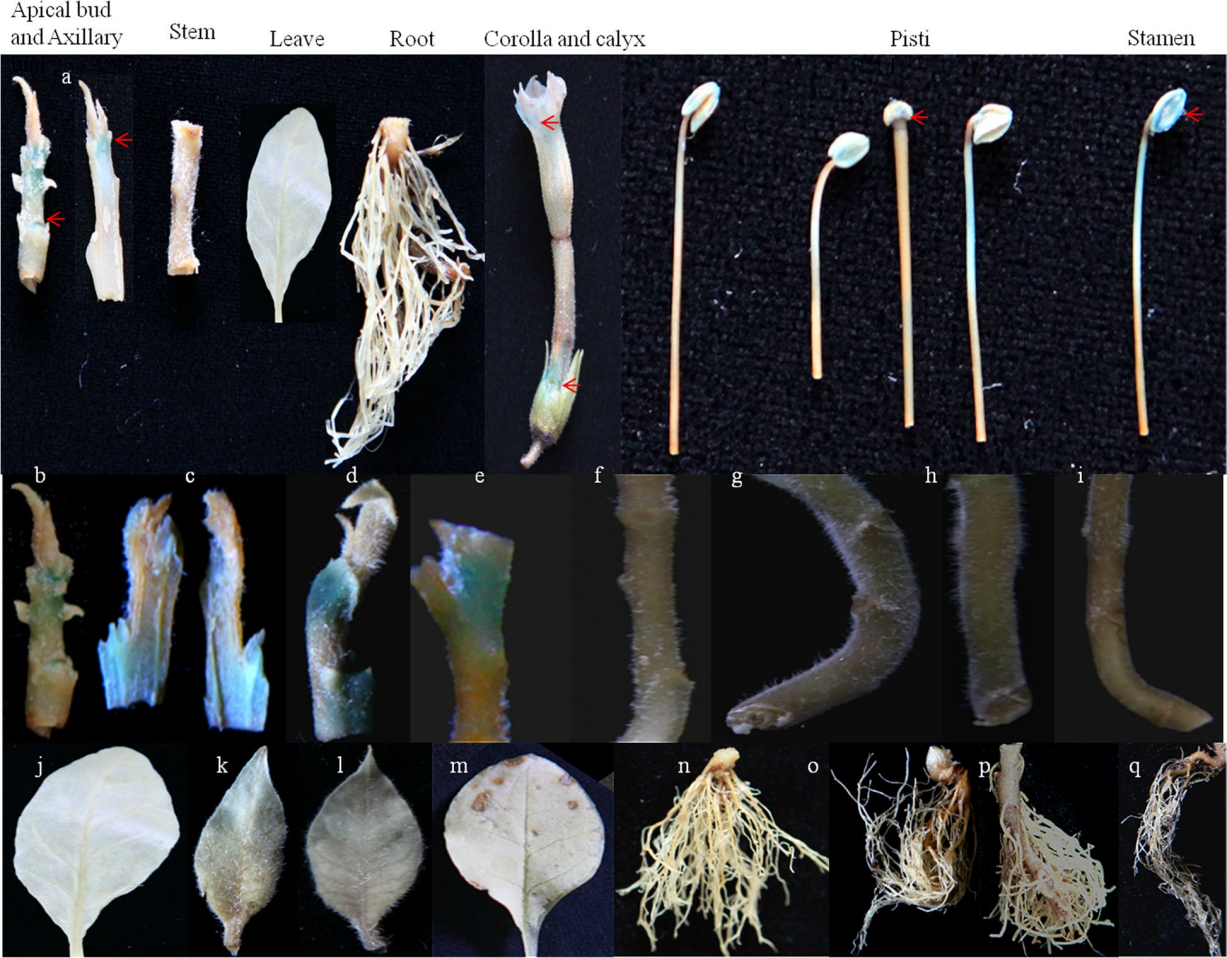
GUS verification of *P*_*NtGDSL127*_ and 5′ delection promoters in transgenic tobacco plants. **a**: GUS staining of P_*NtGDSL127*_ transgenic plants. **b-e**: GUS staining in apical bud/axillary bud of P_*NtGDSL127*,_ P_*NtGDSL127*-A,_ P_*NtGDSL127*-B_, P_*NtGDSL127*-C_ transgenic plants separately. **f-i**: GUS staining in stem of P_*NtGDSL127*,_ P_*NtGDSL127*-A,_ P_*NtGDSL127*-B_, P_*NtGDSL127*-C_ transgenic plants separately. **j-m**: GUS staining in leaf of P_*NtGDSL127*,_ P_*NtGDSL127*-A,_ P_*NtGDSL127*-B_, P_*NtGDSL127*-C_ transgenic plants separately. **n-q**:GUS staining in root of P_*NtGDSL127*,_ P_*NtGDSL127*-A,_ P_*NtGDSL127*-B_, P_*NtGDSL127*-C_ transgenic plants separately. *P*_*NtGDSL127*_ was specifically expressed in apical and axillary buds, as well as in flowers, the 235 bp of *P*_*NtGDSL127*_ is enough to drive the expression of the *GUS* gene

**Fig. 10:**
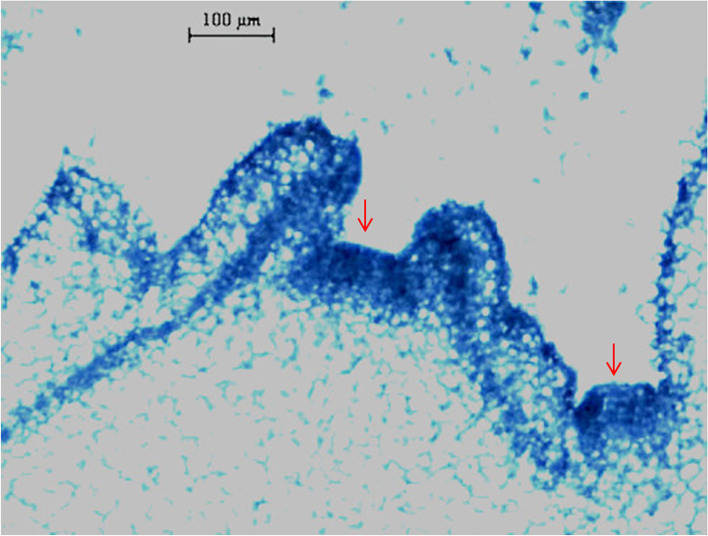
Histochemical GUS staining of P_*NtGDSL127*_ transgenic plants, *Bars* 100 μm

To further identify the core regulatory regions required for expression specificity, three 5′ deletion promoters were constructed and introduced into tobacco (Fig. 8). 22(*P*_*NtGDSL127::*_*GUS*), 25(*P*_*NtGDSL127-A::*_*GUS*), 27(*P*_*NtGDSL127-B::*_*GUS*), and 30(*P*_*NtGDSL127-C::*_*GUS*) independent transformants of each construct were obtained separately. The leaves, stems, roots, flowers, and apical and axillary buds at different stages were used to assess the histochemical expression of GUS. GUS staining results demonstrated that *P*_*NtGDSL127*_ was specifically expressed in apical and axillary buds, as well as in flowers (Fig. 9a, Fig. 10); this result is consistent with the results of qRT-PCR. *P*_*NtGDSL127*_ presented the same expression pattern as *P*_*NtGDSL127-A*_, *P*_*NtGDSL127-B*_*,* and *P*_*NtGDSL127-C*_ in apical buds/axillary buds (Fig. 9b-e), stems (Fig. 9f-i), leaves (Fig. 9j-m) and roots (Fig. 9n-q). These results indicated that 235 bp of *P*_*NtGDSL127*_ is sufficient to drive the expression of the *GUS* gene. Further 5′ deletion promoter and mutation experiments are necessary to determine the core cis-acting element required for meristem-specific expression.

### Phenotypic observation of *P*_*NtGDSL127*_::*DTA* transgenic tobacco

**Fig. 11:**
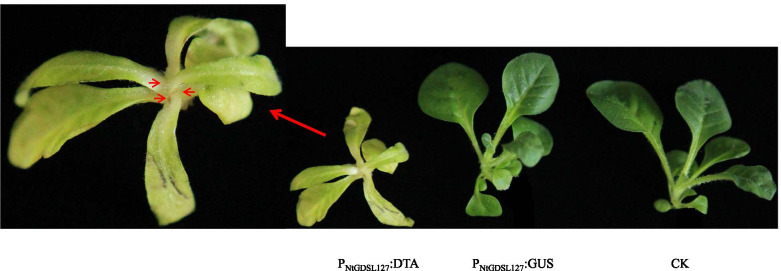
Phenotypic contrast between transgenic materials and controlled plants. The 2132-bp promoter of *NtGDSL127* can drive the expression specific to apical and axillary buds of tobacco

In the current study, the recombinant vector containing *DTA* driven by the 2132-bp *P*_*NtGDSL127*_ was constructed based on the *P*_*NtGDSL127*_::*GUS* recombinant vector. The phenotype of the *P*_*NtGDSL127*_::*DTA* transformants was observed and compared with that of cultivated ‘honghuadajinyuan’ tobacco plants (Fig. 11). The apical and axillary buds of *P*_*NtGDSL127*_::*DTA* transformants were absent, which was consistent with the GUS verification of *P*_*NtGDSL127*_::*GUS* transformants. These results further verified that the 2132-bp promoter of *NtGDSL127* drove the expression specific to apical and axillary buds of tobacco.

## Discussion

GDSL lipases are involved in growth and development, organ morphogenesis, secondary metabolism, and stress resistance in plants, as documented in *Arabidopsis thaliana*, *B. napus*, *Capsicum annuum,* and *Zea mays*. However, the role of *GDSL* genes in tobacco was previously unknown. Here, 159 GDSL proteins in the tobacco genome were identified and divided into three subgroups based on their phylogenetic relationships, which was consistent with the division of the *GDSL* family in other species. Based on the expression profiling of 93 *GDSL* genes before and after topping, the tissue expression patterns of 13 candidate genes that were observably up-regulated or down-regulated were examined by qRT-PCR.

In tobacco field production, the terminal bud or inflorescence is removed from the top to facilitate nutrient transfer to the leaves, in a process known as topping. Topping can release bud dormancy and activate genes associated with bud initiation and development, leading to axillary bud growth [31]. Therefore, the 13 candidate genes screened from transcriptome data that responded to topping are likely involved in the regulation of axillary bud initiation and expressed specifically in axillary buds. The qRT-PCR results demonstrated that one gene, *NtGDSL127*, had tissue specificity to the terminal and axillary buds. The GUS assay indicated that the 2132-bp promoter of *NtGDSL127* was meristem-specific and further supported these findings. The 5′ deletion promoter analysis revealed that a 235-bp promoter was capable of directing meristem-specific expression, indicating that *NtGDSL127* is involved in the regulation of terminal and axillary bud initiation. In addition, *NtGDSL127* responded to hormone applications, suggesting that *NtGDSL127* is involved in the growth and developmental regulation of the apical and axillary buds. These results present a new function of *GDSL* genes in bud regulation in tobacco. Thus, the molecular mechanism whereby *NtGDSL127* regulates the growth and development of the meristem should be further studied.

More GUS recombinant vectors driven by shorter 5′ deletion promoters need to be constructed to determine the core regulatory region and elements that lead to the meristem-specific expression of P_*NtGDSL127*_. Genes with different functions driven by *P*_*NtGDSL127*_ can be identified and used for developmental regulation and ideal plant architecture breeding in tobacco.

## Conclusions

In this study, 159 *GDSL* genes were identified in cultivated tobacco and comprehensive analysis of the *GDSL* gene family was performed, including conserved domain, phylogenetic relationship, gene structure, as well as expression pattern analysis. In addition, a total of 13 *GDSL* genes screened from transcriptome data were shown to substantially up- or down-regulate in response to topping and may be candidate genes involved in the regulation of axillary bud initiation. Moreover, we found that the *NtGDSL127* gene was specifically expressed in apical and axillary buds and flowers in tobacco, which provides further insight for the construction of recombinant vectors containing genes with different functions driven by the *NtGDSL127 promoter* and facilitates tobacco breeding for beneficial morphology.

## Methods

### Plant materials and sample preparation

*Nicotiana tabacum* L. cv. ‘honghuadajinyuan’ was grown in the greenhouse. The seeds were obtained from the Tobacco Research Institute (TRI) of the Chinese Academy of Agricultural Sciences (CAAS). The roots, stems, leaves, flowers, apical and auxiliary buds of the tobacco plants were collected during the vigorous growth period and stored individually at − 80 °C.

### Identification of *GDSL* members in *Nicotiana tabacum*

Previously reported transcriptome data [28] were reannotated using the 2017 tobacco genome (https://solgenomics.net/organism/Nicotiana_tabacum/genome) [32] and reanalyzed. The reannotated tobacco genomic sequences were used for gene identification. The Hidden Markov Model (HMM) profile of the GDSL domain (PF00657) retrieved from Pfam was used to conduct a HMM search against the annotated protein database, with an E-value cutoff of 1.0, using HMMER (v 3.0) [33]. A BLASTP-based search against the 2017 annotations to the tobacco genome was performed to identify each newly identified entry, and redundant hits were removed manually. The resulting sequences were then analysed with both Pfam (https://pfam.xfam.org/) and SMART (http://smart.embl.de/) to ensure the presence of the GDSL domain.

### Multiple sequence alignment and phylogenetic analysis

A multiple sequence alignment of full-length amino acid sequences of putative *GDSL* members from tobacco was performed using MAFFT v5.3 with the default settings [34]. Phylogenetic trees were constructed using the neighbour-joining method based on the alignment results. A unrooted tree was constructed from the alignment of full-length amino acid sequences of *GDSL* members using MEGA v7.0 [35] with the following parameters: Poisson correction, pairwise deletion, and bootstrap values (1000 replicates). Sequence alignment results are presented with ESPript 3.0 (http://espript.ibcp.fr/ESPript/cgi-bin/ESPript.cgi). Protein motifs were predicted by the motif elicitation program MEME (http://meme-suite.org/tools/meme). The isoelectric point and molecular weight of deduced GDSL proteins were predicted by the ProtParam tool (http://web.expasy.org/protparam/).

### Gene expression profiling and selection of candidate genes

The data used for expression profiling of tobacco *GDSL* genes were from the tobacco Illumina HiSeq™ 2000 RNA-seq data [28]. From the reannotated and reanalysed RNA-seq data with the 2017 tobacco genome, FPKM values of 93 *GDSL* genes were retrieved and normalised (Supplementary Table S2). A heatmap was generated based on the log2 fold-change values at TY1/TY3/TY5 when compared with DY and visualised with Cluster3.0 [36] and TreeView [37]. Genes that were up- or down-regulated twofold or more were chosen for subsequent analyses.

### RNA extraction and qRT-PCR

Total RNA from each sample (roots, stems, leaves, flowers, apical and axillary buds) was extracted using the GeneJET™ Plant RNA Purification Mini Kit (MBI Fermentas, Canada). Samples were run on 1% agarose gels, and the purity was checked using a NanoDrop2000 spectrophotometer. Total RNA was reverse transcribed using the RevertAid™ First-Strand cDNA Synthesis Kit (MBI Fermentas, Canada). cDNA was used for qRT-PCR and fluorescence quantitative PCR analyses using TB Green™ Premix Ex Taq™ II (Tli RNaseH Plus) (TaKaRa, Japan) with primers specific to the candidate genes (Table 3).

**Table 3:** Primer sequences of candidate genes used for qRT-PCR

Name	Sequence (5′–3′)	
	F	R
*NtGDSL8*	GGTGGTTTTTGGGCAGCTTT	GAGGCAGCTCCACCAGAAAT
*NtGDSL13*	ATCTCATTGCACAACACTATGG	CCCTAGAAGAAATAATGACCTCTC
*NtGDSL27*	GGGATTCAAATACTGATACTGG	GATTAGTGTTCCCTTTACTCTG
*NtGDSL45*	ACCATACTTCCTGCCACTGC	GGGTCCTCCGTATCCACAAC
*NtGDSL55*	GCTTGTTGCAGGCGTTGGT	TAAGCAACCCAAAGGACCGG
*NtGDSL74*	GGTCACAAGAACAAGAACCC	ATGATCTCCCAAGTTATTTCCC
*NtGDSL75*	TTTAAAGGCAATCACCCACCC	GGCTGTAAATATGGTGGCACA
*NtGDSL79*	GTTTAGTGTATGTGAAGCGAAGG	GTTCGACCGCTATCAAATCC
*NtGDSL89*	TGTACACCAGGTTCTAGGGC	CGTCCCACAACAAGCACTTT
*NtGDSL109*	AGCCGAGAAGCTAGAGGGAA	AGACAACGAGCTTGATGCCA
*NtGDSL110*	GAGAACAGAGCCAATTATGAACC	TCCAGATCAATAACCAAACCAG
*NtGDSL112*	CTCAATTAAGCGGCGTAATCC	CCCTACAACTCCGATCAGTC
*NtGDSL127*	TGTATAGCTATGGAGCAAGGA	CACTGTAAATCCAACAGGTGAG

### Hormone application and fluorescence quantitative PCR

Hormonal treatments of 100 mg/l gibberellic acid (GA3), 0.1 mmol/l abscisic acid (ABA), 0.0001 mol/l indole-3-acetic acid, 1 mg/ml salicylic acid, and 91% methyl jasmonate were separately applied to *N. tabacum* L. cv. ‘honghuadajinyuan’ during the vigorous growth period, and the axillary buds were sampled at four time points (0, 0.5, 2, and 4 h) after hormone application. Three biological replicates were performed for each treatment.

### Isolation and sequence analysis of the *NtGDSL* promoter

The upstream region of the *NtGDSL127* gene was amplified from *N. tabacum* L. cv. ‘honghuadajinyuan’ genomic DNA with PCR using the specific primers P_NtGDSL127_-F and P_NtGDSL127_-R (Table 4). The PCR product was cloned into the pEASY™ T5 Zero vector according to the manufacturer’s protocol (Promega, Madison, WI, USA). The sequenced DNA fragment was designated *P*_*NtGDSL127*_*.* Putative regulatory elements contained within the *P*_*NtGDSL127*_ promoter were analysed using the PLACE database (https://www.dna.affrc.go.jp/PLACE/?action=newplace). To further identify the regulatory regions required for expression specificity, three 5′ deletion promoters (*P*_*NtGDSL127-A*,_ *P*_*NtGDSL127-B*_*,* and *P*_*NtGDSL127-C*_) were cloned (Table 4) into the pEASY™ T5 Zero vector.

**Table 4:** Primer sequences used to amplify promoters

Name	Product Length (bp)	Sequence (5′–3′)	
		F	R
*P*_*NtGDSL127*_	2132	CCCAAGCTTAACGAGGAAAACTAAATGAAC	ACGCGTCGACTCTTGAATTGATTGAAGAAATC
*P*_*NtGDSL127-A*_	1211	CCCAAGCTTGTTGTCTTCGTGTGACCTAT	ACGCCGTCGACTCTTGAATTGATTGAAGAAATC
*P*_*NtGDSL127-B*_	745	CCCAAGCTTGAAGGCTCTCAGTCAAACGA	ACGCCGTCGACTCTTGAATTGATTGAAGAAATC
*P*_*NtGDSL127-C*_	235	CCCAAGCTTGAAGACGAGTAAATAAAAGTA	ACGCCGTCGACTCTTGAATTGATTGAAGAAATC
*GUS*	\	GCCTTGCTAATGGTAATGGTG	TTGACCCACACTTTGCCGTA
*NtActin*		CAAGGAAATCACCGCTTTGG	AAGGGATGCGAGGATGGA

### Construction of *P*_*NtGDSL127*_::*GUS* recombinant vectors and genetic transformation

The 2132-bp genomic fragment (Fig. 4) flanking the 5′ end of *NtGDSL127* and three 5′ deletion promoters were amplified by PCR and inserted separately in-frame in front of the *GUS* reporter gene at the *Hind*III and *Bam*HI restriction enzyme sites in the *PBI121* vector (Fig. 5). These constructs, named *P*_*NtGDSL127*_::*GUS* and *P*_*NtGDSL127-A*_, _*−B*_ and _*-C*_::*GUS*, respectively, were then separately transformed into *Agrobacterium tumefaciens* strain EHA105 and introduced into cultivated *N. tabacum* L. cv. ‘honghuadajinyuan’ plants using the leaf disc method. Transgenic plants were selected on LB media containing 0.1 mg/ml kanamycin.

### Construction of *P*_*NtGDSL127*_*::DTA* recombinant vector and genetic transformation

The CDS sequence of the lethal diphtheria toxin *DTA* gene(GenBank: KY766997.1) was synthesised and inserted into the *P*_*NtGDSL127*_::*GUS* vector using restriction enzymes *Bam*HI and *Sac*I. The recombinant vector, *P*_*NtGDSL127*_::*DTA*, was transformed into *A. tumefaciens* strain EHA105 and introduced into cultivated *N. tabacum* L. cv. ‘honghuadajinyuan’ plants using the leaf disc method. Transgenic plants were selected on LB media containing 0.1 mg/ml kanamycin.

### GUS staining, tissue processing, and microscopic observations

Positive transgenic plants were selected by PCR using the specific primers GUS-F and GUS-R (Table 4). Representative tissues of transgenic tobacco were sampled for GUS histochemical staining, as previously described [38]. After incubation in GUS staining solution overnight at 37 °C, the samples were successively decoloured in 70, 85, and 100% ethanol until the chlorophyll pigments were completely removed. Samples were fixed with formalin:acetic acid:50% ethanol (1:1:18, v/v/v) for at least 24 h. After fixation, samples were observed and photographed (Nikon ECLIPSE 80i, Japan). After GUS staining, the samples were observed in paraffin sections. The samples were dehydrated in an ethanol series, and infiltrated with xylene followed by paraffin, then embedded in paraffin using a Leica Paraffin-Embedder (Leica Microsystems Inc., Deerfield, IL) [39], observed, and photographed (Nikon ECLIPSE 80i, Japan).

## Supplementary Information

**Additional file 1: Table S1.** Basic information of *GDSL* gene family in *Nicotiana tabacum*.

**Additional file 2: Table S2.** Candidate genes and the transcriptome data.

**Additional file 3: Table S3.** The sequences of the *GDSL* family members in tobacco.

## Data Availability

All data generated or analysed during this study are included in this published article and its supplementary information files, the sequencing data analyzed in this study has been uploaded in the NCBI SRA database(SRA:SRP269197).
